# Determining the oxidation state of elements by X-ray crystallography

**DOI:** 10.1107/S2059798321013048

**Published:** 2022-01-24

**Authors:** Frank Lennartz, Jae-Hun Jeoung, Stefan Ruenger, Holger Dobbek, Manfred S. Weiss

**Affiliations:** a Macromolecular Crystallography, Helmholtz-Zentrum Berlin, Albert-Einstein-Strasse 15, 12489 Berlin, Germany; b Department of Biology, Humboldt-Universität zu Berlin, 10099 Berlin, Germany

**Keywords:** SpReAD, spatially resolved anomalous dispersion refinement, radiation damage, photoreduction, redox reactions, metalloproteins, oxidoreductases, sulerythrin

## Abstract

Protein-mediated redox reactions play a critical role in many biological processes and often occur at centres that contain metal ions as cofactors. In order to understand the exact mechanisms behind these reactions it is important to not only characterize the three-dimensional structures of these proteins and their cofactors, but also to identify the oxidation states of the cofactors involved and to correlate this knowledge with structural information. The only suitable approach for this based on crystallographic measurements is spatially resolved anomalous dispersion (SpReAD) refinement, a method that has been used previously to determine the redox states of metals in iron–sulfur cluster-containing proteins. In this article, the feasibility of this approach for small, non-iron–sulfur redox centres is demonstrated by employing SpReAD analysis to characterize *Sulfolobus tokodaii* sulerythrin, a ruberythrin-like protein that contains a binuclear metal centre. Differences in oxidation states between the individual iron ions of the binuclear metal centre are revealed in sulerythrin crystals treated with H_2_O_2_. Furthermore, data collection at high X-ray doses leads to photoreduction of this metal centre, showing that careful control of the total absorbed dose is a prerequisite for successfully determining the oxidation state through SpReAD analysis.

## Abstract 2

Using spatially resolved anomalous dispersion (SpReAD) refinement, the oxidation states of elements in crystal structures can be determined.

## 1. Introduction

Current estimates suggest that 30–50% of all proteins bind metal ions and, indeed, more than 35% of all structures currently deposited in the Protein Data Bank are of such metalloproteins (Waldron *et al.*, 2009 ▸; Putignano *et al.*, 2018 ▸). Metalloproteins can mediate complex chemical reactions through their metal cofactors, including essential biological processes such as respiration and photosynthesis. The metals coordinated by these proteins can be isolated ions or parts of more complex cofactors such as haem groups or iron–sulfur clusters (Harding *et al.*, 2010 ▸). They often are first-row transition metals, including iron, zinc, manganese and copper, which play key roles in structure stabilization, oxygen and lipid metabolism, detoxification of reactive oxygen species, DNA replication and electron transport (Waldron *et al.*, 2009 ▸; Bowman *et al.*, 2016 ▸). Some metalloproteins also mediate oxidation–reduction reactions, during which their metal cofactors undergo complex redox reactions, for example in cytochromes or iron–sulfur proteins, where metal ions are directly involved in electron transfer (Liu *et al.*, 2014 ▸). In order to understand the exact mechanism of reactions catalysed by proteins harbouring such redox-active centres, it is crucial not only to know the 3D structures of the protein and the catalytic site, but also to assign the redox state of its cofactor.

While X-ray crystallography is typically used to determine the structures of these proteins, the resulting electron-density maps themselves do not normally reveal the oxidation states of individual metal ions. Additional experiments are therefore often necessary to identify the redox states of metals in metalloproteins and to correlate this information with the structure determined by X-ray crystallography. Methods commonly used for this include electron paramagnetic resonance (EPR) spectroscopy, which is limited to paramagnetic metals, and X-ray absorption spectroscopy, such as X-ray absorption near-edge structure (XANES) and extended X-ray absorption fine structure (EXAFS) (Gambarelli & Maurel, 2014 ▸; Ward *et al.*, 2014 ▸). These techniques are integrative and lack the element of spatial resolution (Ward *et al.*, 2014 ▸). They therefore offer no straightforward way to assign oxidation states to individual metal sites, which is often crucial for functional analysis and for the understanding of the structure–function relationship of these sites. One potential approach to obtain this information by X-ray crystallography is based on the fact that the lengths of metal–ligand bonds are sensitive to the oxidation state of the metal. The drawback of this method is not only that it requires a resolution high enough to unambiguously assign the position of the metal and its ligands, but also that even within the same oxidation state bond lengths can considerably vary due to the spin state or the geometry of the coordination sphere (Zheng *et al.*, 2008 ▸, 2017 ▸).

A method that provides a solution to this is spatially resolved anomalous dispersion (SpReAD) refinement (Einsle *et al.*, 2007 ▸). SpReAD takes advantage of the spatial resolution of X-ray crystallographic data and the observation that the position of an X-ray absorption edge of elements such as transition metals is sensitive to their redox state, with higher oxidation states resulting in edges shifted to a higher energy due to the higher energy needed to remove a core electron (Spatzal *et al.*, 2016 ▸). In SpReAD refinement, Δ*f*′′ values for individual metal atoms are calculated by refining the structure against X-ray data collected at different energies across a transition-metal absorption edge, typically in steps of 2 eV. From these values, absorption spectra for each individual metal site can be reconstructed. The relative positions of their inflection points then indicate the oxidation states of the respective metals. This approach has been used to determine the oxidation states of individual metals in complex metal centres, such as those found in different iron–sulfur cluster-containing proteins such as *Aquifex aeolicus* ferredoxin, the *Azotobacter vinelandii* MoFe and Fe proteins, and the iron–molybdenum cofactor of nitrogenase (Spatzal *et al.*, 2016 ▸; Einsle *et al.*, 2007 ▸; Zhang *et al.*, 2013 ▸; Wenke *et al.*, 2019 ▸), but it can be used on any protein that contains a metal cofactor.

One such protein is sulerythrin (SulE), a ruberythrin-like protein isolated from the thermophilic archeon *Sulfolobus tokodaii*. SulE lacks the characteristic C-terminal, rubredoxin-like FeS_4_ domain of ruberythrins and contains a binuclear metal centre that coordinates iron and zinc when isolated from *S. tokodaii* (Wakagi, 2003 ▸). In contrast to other members of the ruberythrin family, a crystal structure of native SulE shows that it forms homodimers through domain swapping, with each monomer contributing two α-helices to give two four-helix bundles that each harbour a solvent-accessible, single dimetal site (Fushinobu *et al.*, 2003 ▸). The exact function of SulE is unknown, but it is presumed to be involved in reactions against oxidative stress (Wakagi, 2003 ▸).

Here, SulE reconstituted with ferrous iron (diFe-SulE) and treated with H_2_O_2_ was used as a model for SpReAD analysis using data collected on beamline BL14.1 at the Helmholtz-Zentrum Berlin (Mueller *et al.*, 2012 ▸, 2015 ▸). The experiment shows that each monomer in diFe-SulE coordinates two iron ions and that one of these is in a more reduced state, whereas the other iron ion is in a more oxidized state. This study further demonstrates that the observed oxidation state is sensitive to X-ray-induced photoreduction and that data collection at high total doses leads to partial reduction of the more oxidized iron. This in turn highlights the need for careful design of the diffraction experiment for SpReAD analysis, in particular with respect to keeping the total absorbed dose during data collection to a minimum.

## 2. Materials and methods

### 2.1. Expression, purification and iron reconstitution of *Sulfolobus tokodaii* SulE

A synthetic gene encoding SulE (UniProt accession No. F9VPE5, residues 1–145) was cloned into the pET-28a expression vector in frame with the sequence for an N-terminal Strep-tag followed by a Tobacco etch virus (TEV) protease cleavage site, as described by Jeoung *et al.* (2021 ▸). The protein was expressed in *Escherichia coli* BL21 (DE3) cells grown in minimal medium (Dydio *et al.*, 2016 ▸) with 50 µg ml^−1^ kanamycin at 37°C. Expression was induced with 0.5 m*M* isopropyl β-d-1-thiogalactopyranoside at an optical density (OD_600_) of 0.4. After induction, the cells were further cultivated for 22 h at 25°C. The cells were then harvested by centrifugation, resuspended in 50 m*M* Tris–HCl pH 7.5, 150 m*M* NaCl, disrupted by sonication in the presence of avidin, lysozyme and DNaseA and centrifuged for 45 min at 35 000*g*. SulE was purified from the supernatant by affinity chromatography using Strep-Tactin Superflow high-capacity resin (IBA, Göttingen, Germany). The Strep-tag was removed from the purified protein by overnight incubation at 25°C with Strep-tagged TEV protease in the presence of 10 m*M* β-mercapto­ethanol. TEV protease was removed the next day by affinity chromatography using Strep-Tactin Superflow high-capacity resin. SulE was further purified by size-exclusion chromatography using a Superdex 200 column (Cytiva) in buffer consisting of 50 m*M* Tris–HCl pH 7.5, 150 m*M* NaCl. Fractions containing pure SulE, as verified by sodium dodecyl sulfate polyacrylamide gel electrophoresis, were further concentrated and frozen at −80°C until further use. Reconstitution of SulE with iron was performed under anoxic conditions in an atmosphere of 95% N_2_/5% H_2_ inside a glove box (Coy Laboratory Products). 520 µ*M* apo SulE was mixed with 2 m*M* ascorbic acid and 1.2 m*M* FeSO_4_ and incubated at 18°C for 3 h. Unbound metal was then removed by repeated cycles of diluting and concentrating the sample using a spin concentrator.

### 2.2. Crystallization, crystal treatment and cryocooling

All crystallization experiments were conducted inside a glove box (Coy Laboratory Products) under anoxic conditions in an atmosphere of 95% N_2_/5% H_2_. For crystallization, purified SulE reconstituted with iron was concentrated to 14 mg ml^−1^. Crystals were grown using the vapour-diffusion method in sitting drops by mixing 0.5 µl protein solution with 0.5 µl well solution. Initial crystals grew at 293 K in 0.1 *M* bis-Tris pH 5.5, 25%(*w*/*v*) PEG 3350. Crystallization conditions were further optimized by modifying the concentration of PEG, and larger crystals grew in 0.1 *M* bis-Tris pH 5.5, 24%(*w*/*v*) PEG 3350. For treatment with H_2_O_2_, individual crystals were harvested, transferred into a drop containing well solution supplemented with 100 m*M* H_2_O_2_ and incubated for >2 min until a colour change was visible. After this, the crystals were transferred into a drop of well solution supplemented with 25%(*v*/*v*) glycerol for cryoprotection, incubated for 5 s and then flash-cooled in liquid nitrogen. For further details, see Jeoung *et al.* (2021 ▸).

### 2.3. Data collection and processing

All diffraction data were collected on the BESSY II macromolecular crystallography beamline BL14.1 (Gerlach *et al.*, 2016 ▸; Mueller *et al.*, 2012 ▸, 2015 ▸) using a PILATUS3 S 6M detector (Dectris) and a 50 µm aperture. The crystals were mounted on a MD2 microdiffractometer with mini-kappa goniometer and cooled to 100 K in a nitrogen gas stream. For each crystal and beamline flux setting, a reference data set was collected in steps of 0.1° over 220° with 0.1 s exposure per frame at an energy of 13.5 keV. The metal content of all crystals was analyzed by recording X-ray fluorescence spectra at 13.5 keV and the spectra were inspected with *XFEplot* (https://www.helmholtz-berlin.de/forschung/oe/ps/macromolecular-crystallography/hzb-mx-software/xfeplot/index_en.html). The exact position of the Fe *K* absorption edge was determined via an X-ray absorption-edge scan. Nine data sets over 200° in steps of 0.1° with 0.1 s exposure per frame were then collected in steps of 2 eV at energies across the Fe *K* edge, with the first data set collected at 7114 eV and the last data set collected at 7130 eV. The individual data sets were processed using *XDSAPP* (Sparta *et al.*, 2016 ▸), keeping the Friedel pairs separate. When processing the data collected across the Fe *K* edge, the unit-cell dimensions were fixed to the values obtained from processing the reference data set. To allow better comparison of data-quality indicators between these data sets, the highest resolution shell was set to a fixed limit of 1.87 Å. The individual data sets collected across the Fe *K* edge were then scaled against each other using *XSCALE* (Kabsch, 2010 ▸).

For a single crystal, this procedure was conducted at different beamline flux settings of 5%, 10% and 100% transmission, corresponding to 1.67 × 10^9^, 2.73 × 10^9^ and 4.25 × 10^10^ photons s^−1^ at the sample position at 13.5 keV with the 50 µm aperture used here. For each of these settings, a reference data set and data sets across the Fe *K* edge were collected from individual, non-overlapping positions of the crystal. Statistics for the different data collections are shown in Table 1 ▸.

### 2.4. Structure solution and refinement

The high-resolution structures of diFe-SulE were solved by molecular replacement with *Phaser-MR* (McCoy *et al.*, 2007 ▸), using the reference data sets collected at 13.5 keV and a model of SulE (PDB entry 1j30; Fushinobu *et al.*, 2003 ▸) with all metal atoms omitted as a template. For iron-ion placement, anomalous difference density maps were calculated with *phenix.refine* (Liebschner *et al.*, 2019 ▸; Afonine *et al.*, 2012 ▸) using data sets collected at the Fe *K*-edge peak energy of 7126 eV. Missing parts of the model were built by iterative cycles of refinement with *phenix.refine*, using automatically generated noncrystallographic symmetry and translation/libration/screw restraints, and model building in *Coot* (Emsley *et al.*, 2010 ▸). H atoms at riding positions were added during refinement. All Fe atoms were refined with individual anisotropic atomic displacement parameters and their occupancy was refined in the last refinement step. Statistics for structure solution and refinement are reported in Table 2 ▸. Composite omit electron-density maps were calculated with *Phenix* (Liebschner *et al.*, 2019 ▸). All structure figures were prepared using *PyMOL* (Schrödinger).

### 2.5. Spatially resolved anomalous dispersion (SpReAD) analysis

SpReAD refinement takes advantage of the spatial resolution of diffraction data sets and is based on the refinement of the anomalous scattering contributions *f*′ and *f*′′ for each individual heavy atom of interest. The detailed theoretical background for the method has been described previously (Einsle *et al.*, 2007 ▸). For SpReAD analysis of diFe-SulE, the values of *f*′ and *f*′′ for individual Fe atoms were refined against the anomalous differences for individual structure factors using *phenix.refine*. All four Fe atoms in the asymmetric unit were included in this refinement. The model refined against the reference data set was used to calculate the phases. This was repeated for each individual data set across the Fe *K* edge and for each beamline flux setting, and the resulting *f*′′ values were then plotted against the energy.

### 2.6. Dose estimation

The total absorbed dose for each crystal position and at each beamline flux setting was estimated using *RADDOSE*-3*D* (Zeldin *et al.*, 2013 ▸; Bury *et al.*, 2018 ▸). This was performed for each individual measurement, factoring in the respective energy, total exposure time and measured wedge, as described in Table 1 ▸. The crystal input parameters for *RADDOSE*-3*D* were derived from measuring the size of the crystal and the processing results for the reference data sets. The solvent fraction was derived from the Matthews coefficient. The incident photon flux at the sample position used as input for *RADDOSE*-3*D* was derived as follows: the incident X-ray intensities were monitored using a nitrogen-filled ionization chamber, and the measured value was used to calculate the photon flux based on a calibration curve created using an X-ray photodiode. All other parameters were set to the defaults. The values reported here are average diffraction-weighted doses.

### 2.7. Accession numbers

The refined coordinates and the structure-factor amplitudes have been deposited in the Protein Data Bank under the accession numbers 7ppt for the data collected at 0.26 MGy, 7ppu for the data collected at 0.57 MGy and 7ppv for the data collected at 2.70 MGy total absorbed dose. The raw diffraction images have been deposited in the Integrated Resource for Reproducibility in Macromolecular Crystallo­graphy (Grabowski *et al.*, 2016 ▸; http://proteindiffraction.org/) and are accessible via the respective PDB entries.

## 3. Results

### 3.1. The overall structure of diFe-SulE

**Figure 1:**
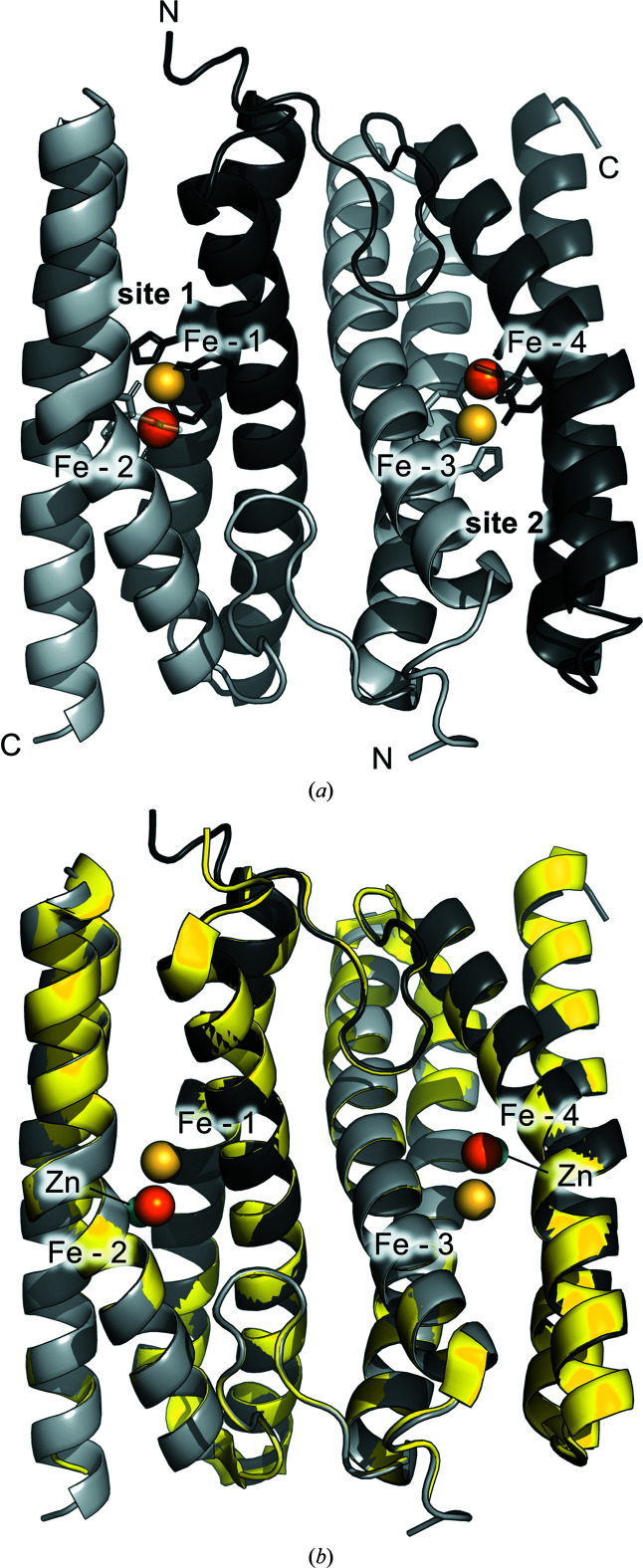
Overall structure of diFe-SulE. (*a*) DiFe-SulE is shown in ribbon representation, with colours indicating the two chains (black, chain *A*; grey, chain *B*) of the homodimer in the asymmetric unit. The two binuclear metal sites are labelled site 1 and site 2. The four iron ions are shown as spheres and are labelled Fe-1 to Fe-4. Residues that coordinate the iron ions are shown as sticks and are coloured by chain. The N- and C-­termini of the two chains are indicated. (*b*) Superposition of diFe-SulE (black/grey) with Fe/Zn-SulE (yellow; PDB entry 1j30). The Zn atom (teal) that occupies the position of Fe-2 and Fe-4 in Fe/Zn-SulE is indicated. The superposition was prepared with *PyMOL*.

**Figure 2:**
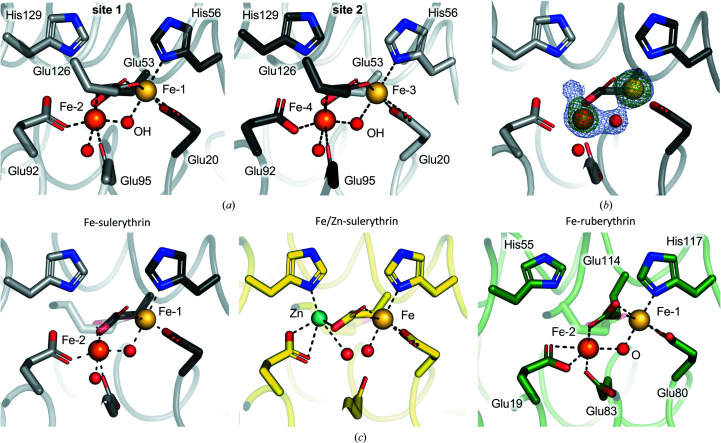
Coordination of the Fe atoms in the binuclear metal-binding sites of diFe-SulE. (*a*) Details of the two binuclear metal-binding sites. Site 1 is shown on the left and site 2 on the right. Residues involved in the coordination of Fe-1 to Fe-4 are shown in stick representation and coloured by atom type. Fe-1 to Fe-­4 are shown as spheres and coloured as in Fig. 1 ▸. (*b*) A 2*F*_o_ − *F*_c_ composite omit electron-density map (blue mesh) contoured at 2.5σ for Fe-1, Fe-2 and the bridging ligand at metal-binding site 1, overlaid with an anomalous difference electron-density map (green mesh) contoured at 13σ for Fe-1 and Fe-2. For clarity, Glu126 has been omitted. (*c*) Comparison between the coordination of the metal ligands in diFe-SulE (black/grey), Fe/Zn-SulE (PDB entry 1j30; yellow) and *Pyrococcus furiosus* Fe-ruberythrin treated with H_2_O_2_ (PDB entry 3mps; green). Metal ions are shown as spheres. The numbering for both SulE structures corresponds to Fig. 2 ▸(*a*).

Diffraction data from a crystal of recombinantly expressed SulE reconstituted with iron (diFe-SulE) were collected on beamline BL14.1 at the BESSY II electron-storage ring operated by the Helmholtz-Zentrum Berlin (HZB). The beamline flux was set to 5% to minimize radiation-induced damage to the metal centres for initial characterization. The crystal diffracted to 1.42 Å resolution and belonged to space group *P*6_3_ (Table 1 ▸). The structure was determined by molecular replacement (Fig. 1 ▸ *a*) using SulE bound to zinc and iron (Fe/Zn-SulE; PDB entry 1j30; Fushinobu *et al.*, 2003 ▸) as the search model. One diFe-SulE homodimer per asymmetric unit was found in the crystals (Table 2 ▸). The homodimer consists of two four-helix bundles. Each monomer contributes two helices to each of these bundles via domain swapping (Fig. 1 ▸ *a*). Overall, the structure of diFe-SulE is very similar to that of Fe/Zn-SulE purified directly from its source organism *S. tokodaii*, with an overall root-mean-square deviation of 0.17 Å for all backbone C^α^ atoms (Fig. 1 ▸ *b*). A single homodimer contains two metal-binding sites, which coordinate two iron ions each to give a total of four iron ions: Fe-1 to Fe-4 (Fig. 1 ▸ *a*). Each iron ion is coordinated by side chains from both monomers in the homodimer (Fig. 1 ▸ *a*), with no differences between the two metal-binding sites (Fig. 2 ▸ *a*).

### 3.2. Structure of the metal-binding sites in diFe-SulE

An anomalous difference electron-density map shows strong peaks for all four of the iron ions, with slightly stronger peaks for Fe-1 and Fe-3, indicating that these are bound more tightly than Fe-2 and Fe-4 (Fig. 2 ▸ *b*). Indeed, the composite omit and anomalous difference electron-density maps for Fe-2 and Fe-4 show nonspherical density stretched out towards His129 (Fig. 2 ▸ *b*), and the occupancies of Fe-2 and Fe-4 refine to 0.80 and 0.90, respectively, compared with 1.0 for both Fe-1 and Fe-3. This suggests partial movement of Fe-2 and Fe-4, which could either be due to incomplete reaction with H_2_O_2_ or be induced by exposure to radiation.

The two iron ions in each metal-binding site are connected through a bridging ligand (Figs. 2 ▸ *a* and 2 ▸ *b*). Based on the colour change observed in the crystals, the position of the two iron ions and the proposed reaction mechanism between ruberythrin or ruberythrin-like proteins and H_2_O_2_ (Dillard *et al.*, 2011 ▸), this ligand could be an oxide or hydroxide ion. Here, the bridging ligand was tentatively modelled as a hydroxide ion. Fe-1 and Fe-3 are coordinated by Glu20, Glu53 and His56 from monomer 1 and Glu126 from monomer 2, as well as the bridging hydroxide ion (Table 3 ▸, Fig. 2 ▸ *a*). This is very similar to the coordination sphere of these two iron ions in Fe/Zn-SulE isolated from *S. tokodaii* (Fig. 2 ▸ *c*; Fushinobu *et al.*, 2003 ▸). As the coordination of Fe-1 and Fe-3 by Glu20 is symmetrical bidentate, it may be counted as a single coordination (Harding, 1999 ▸), resulting in a distorted trigonal bipyramidal coordination sphere (Table 3 ▸). Fe-2 and Fe-4 are coordinated by Glu53 from monomer 1, Glu92 and Glu126 from monomer 2 and a water molecule as well as the bridging hydroxide ion, resulting in an octahedral geometry for the coordination sphere (Table 3 ▸, Fig. 2 ▸ *a*). Interestingly, this position of Fe-2 and Fe-4 is different from the coordination and position of the equivalent zinc ions in Fe/Zn-SulE, and is comparable to the position of the equivalent iron ions in the mixed-valence state of ruberythrin treated with H_2_O_2_ (Fig. 2 ▸ *c*; Dillard *et al.*, 2011 ▸). Whether Fe-2 and Fe-4 are in the reduced or the oxidized state, however, is not apparent from their position or the electron-density maps alone.

### 3.3. SpReAD analysis of diFe-SulE and the effect of the total absorbed dose

In order to identify the oxidation states of all four iron ions bound in diFe-SulE, nine diffraction data sets were collected in steps of 2 eV across the Fe *K* edge, starting at an energy of 7114 keV. The beamline flux for this data collection was set to 5% to minimize any potential radiation-induced reduction of the metals. SpReAD analysis was then conducted by refining the individual anomalous *f*′′ contribution for each of the four iron ions, using the model refined against data collected at 13.5 keV to calculate the phases. The *f*′′ contributions were used to determine the oxidation states because the *f*′ contributions tend to be highly correlated with the occupancy values and the ADPs of the respective metal ions, whereas the *f*′′ contributions are more independent and hence more robust (Einsle *et al.*, 2007 ▸).

**Figure 3:**
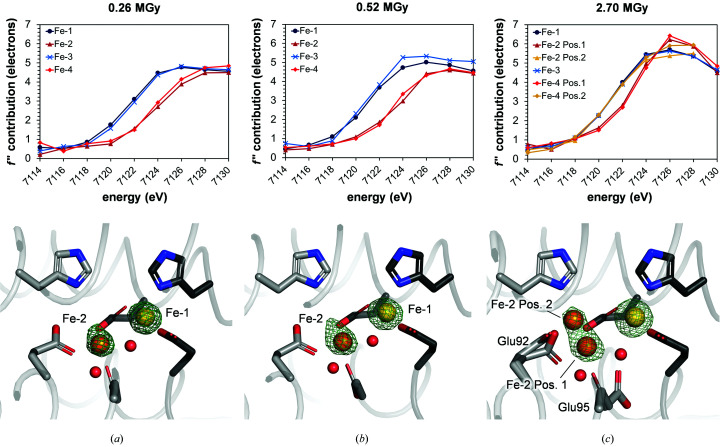
SpReAD analysis of diFe-SulE with data collected at different total average diffraction-weighted doses of (*a*) 0.26 MGy, (*b*) 0.57 MGy and (*c*) 2.70 MGy. The top panel shows the respective SpReAD profiles and the bottom panel shows an anomalous difference electron-density map (green mesh) contoured at 13σ for the data set at 7126 eV. Metal ions are shown as spheres. Residues that show multiple conformations are indicated. For clarity, Glu126 has been omitted.

The resulting SpReAD profiles for each individual iron ion show two populations, with clear differences between Fe-1/Fe-3 and Fe-2/Fe-4 (Fig. 3 ▸ *a*). The edges for Fe-2 and Fe-4 are shifted to higher energies by 2 eV compared with Fe-1 and Fe-3. This indicates that Fe-2 and Fe-4 are in a more oxidized state than Fe-1 and Fe-3 and are likely to be ferric irons, based on the observation that an increase in oxidation leads to an edge shift of between 1 and 5 eV in model iron compounds (Musgrave *et al.*, 1998 ▸; Shulman *et al.*, 1976 ▸).

SpReAD analysis requires the collection of several diffraction data sets across the absorption edge of the element in question, thereby exposing the crystal to an increased radiation dose, which could potentially lead to photoreduction of metal centres (Carugo & Carugo, 2005 ▸). In order to test the effect of the total absorbed dose on SpReAD analysis, data collection at higher flux values was repeated on the same crystal used at 5% beamline flux, choosing distinct, non-overlapping positions at least 50 µm apart. Data sets at 13.5 keV and nine energies over the rising Fe *K* edge were collected at 10% and 100% flux (Table 1 ▸). Using *RADDOSE*-3*D* (Bury *et al.*, 2018 ▸; Zeldin *et al.*, 2013 ▸), the total average diffraction-weighted dose was calculated to be 0.26 MGy for 5% flux, 0.52 MGy for 10% flux and 2.70 MGy for 100% flux. The data collected at 13.5 keV were used for model building (Table 2 ▸) and anomalous difference density maps were used to assign the positions of Fe-1 to Fe-4. While no significant difference in iron position was observed between measurements taken at 5% and 10% beamline flux, the clearly non­spherical anomalous difference density map for data collected at 100% flux shows two maxima, indicating that Fe-2 and Fe-4 partially move 1.9 Å towards His129 (Figs. 3 ▸ *a*–3 ▸ *c*). Furthermore, Glu92 and Glu95 that coordinate Fe-2 and Fe-4 adopt additional side-chain conformations that are consistent with the coordination of Fe-2 and Fe-4 in the two locations (Fig. 3 ▸ *c*). Fe-2 and Fe-4 were therefore modelled in these distinct positions, with occupancies refining to 0.32 and 0.34, respectively, for position 2 close to His129 and 0.68 and 0.66, respectively, for position 1 equivalent to that occupied in the maps calculated from data at 5% and 10% flux.

The SpReAD analysis was then repeated for the data collected at 10% and 100% beamline flux, refining the *f*′′ contribution for each of the two Fe-2 and Fe-4 positions observed at 100% flux separately. While there was no difference in the oxidation states of all iron ions between 5% and 10% flux, at 100% flux a clear difference was visible for Fe-2 and Fe-4 at position 2, where the edge position was coincident with Fe-1 and Fe-3, suggesting that iron ions at this position are reduced (Fig. 3 ▸ *c*). Furthermore, while the edge positions of Fe-2 and Fe-4 at position 1 still indicate a higher oxidation state than Fe-1 and Fe-3, they are shifted towards lower energies, indicating partial reduction of the iron ions at this position (Fig. 3 ▸ *c*).

## 4. Discussion

Identifying the oxidation states of metals in metalloproteins, and correlating this knowledge with structural information, is an essential but challenging step in understanding the reactions catalysed by these proteins. While methods such as EPR, XANES or EXAFS can be used with crystalline samples, they require additional experiments or specialized equipment or lack the spatial resolution needed to differentiate between individual atoms. The results presented in this study highlight SpReAD analysis as an alternative to these methods that allows the determination of the oxidation states of individual atoms by X-ray crystallography. A prerequisite to obtaining data suitable for SpReAD analysis is a tuneable beamline with an energy resolution of 2 eV or less, and this study shows that such data can be collected on the BESSY II MX beamline BL14.1 with no additional sample preparation or equipment. Indeed, collecting data sets at nine energies across the Fe *K* edge and at 13.5 keV took less than 90 min.

In this study, X-ray crystallography and SpReAD analysis were used to assess the oxidation states of individual iron ions in diFe-SulE. The structure obtained for diFe-SulE treated with H_2_O_2_ shows that the two iron ions in each metal-binding site are at different positions (Fig. 2 ▸). Interestingly, this difference is very similar to the redox-dependent positions of the two iron ions in the mixed-valence or diferric state of *Pyrococcus furiosus* ruberythrin (Dillard *et al.*, 2011 ▸), suggesting that the diFe-SulE structure presented here corresponds to either of these states. While the position of Fe-2 and Fe-4 alone does not allow any differentiation between these two possibilities, our analysis revealed that Fe-2 and Fe-4 are in a more oxidized state than Fe-1 and Fe-3. This indicates that the structure observed here is likely to correspond to the mixed-valence state, and highlights the usefulness of the SpReAD method to identify such intermediate reaction states. While the data sets analysed here had a relatively high resolution of 1.87 Å, it is worth noting that SpReAD analysis should give equally good results with data collected to lower resolution, since the only parameters that are refined during SpReAD analysis are the dispersive and anomalous differences. A SpReAD analysis using the data collected at 0.26 MGy total absorbed dose, cut to a high-resolution limit of 3.5 Å, shows that this is indeed the case (data not shown).

SpReAD analysis requires the collection of several data sets across an X-ray absorption edge, and when conducted at modern synchrotron beamlines with bright X-ray beams, such as the BESSY II MX beamline BL14.1, this can alter the oxidation state of redox-active centres (Carugo & Carugo, 2005 ▸). This alteration is caused by the X-ray-induced generation of photoelectrons throughout the exposed crystal, which even at 100 K can move from their point of origin and change the oxidation state of metal centres that they interact with (Beitlich *et al.*, 2007 ▸). Such photoreduction during crystallographic data collection has been observed for many metalloproteins (Beitlich *et al.*, 2007 ▸; Frankaer *et al.*, 2014 ▸; Hersleth & Andersson, 2011 ▸) and is a potentially significant problem for a method that aims to determine the oxidation states of individual elements by X-ray crystallography. Indeed, the total dose of 2.9 MGy taken up by the crystal at 100% beamline flux is close to doses that lead to photoreduction in other metalloproteins, such as insulin or heme-containing proteins (Frankaer *et al.*, 2014 ▸; Pfanzagl *et al.*, 2020 ▸). The study presented here shows that data-collection parameters that lead to a high total absorbed dose indeed result in a partial reduction of the metal in question, and that this is not only visible in the corresponding electron density but also in the SpReAD profiles (Fig. 3 ▸). Such a radiation-induced change can obfuscate the true oxidation state that is present in unirradiated crystals. This is especially important for potential intermediate reaction states that are captured in crystals, such as that observed here, which are highly sensitive to radiation-dependent changes in oxidation state. It is therefore important to note that data for SpReAD analysis should be collected at the lowest dose possible in order to minimize these effects and allow clear, experimental assignment of the redox state. This will be particularly crucial for the usage of computer programs that rely on such data to predict the structures of metallo­proteins, metal ion-binding sites or potentially even the oxidation states of metal cofactors, such as for instance that published by Lin *et al.* (2016 ▸). Clearly, careful experimental identification of the redox state, for example through a SpReAD analysis, will continue to be highly relevant.

## Supplementary Material

PDB reference: SulE at 0.26 MGy, 7ppt

PDB reference: at 0.57 MGy, 7ppu

PDB reference: at 2.70 MGy, 7ppv

## Figures and Tables

**Table:** (*a*) 5% transmission (0.26 MGy total absorbed dose).

	13500 eV	7114 eV	7116 eV	7118 eV	7120 eV	7122 eV	7124 eV	7126 eV	7128 eV	7130 eV
Diffraction source	BL14.1, BESSY II									
Detector	PILATUS3 S 6M									
Temperature (K)	100									
Wavelength (Å)	0.9184	1.74282	1.74233	1.74184	1.74135	1.74086	1.74037	1.73988	1.73939	1.73891
Crystal-to-detector distance (mm)	211	141	141	141	141	141	141	141	141	141
Rotation range per image (°)	0.1	0.1	0.1	0.1	0.1	0.1	0.1	0.1	0.1	0.1
Total rotation range (°)	220	200	200	200	200	200	200	200	200	200
Exposure time per image (s)	0.1	0.1	0.1	0.1	0.1	0.1	0.1	0.1	0.1	0.1
Resolution (Å)	38.54–1.42 (1.51–1.42)	38.50–1.87 (1.92–1.87)	38.50–1.87 (1.92–1.87)	38.50–1.87 (1.92–1.87)	38.50–1.87 (1.92–1.87)	38.50–1.87 (1.92–1.87)	38.50–1.87 (1.92–1.87)	38.50–1.87 (1.92–1.87)	38.50–1.87 (1.92–1.87)	38.50–1.87 (1.92–1.87)
Space group	*P*6_3_	*P*6_3_	*P*6_3_	*P*6_3_	*P*6_3_	*P*6_3_	*P*6_3_	*P*6_3_	*P*6_3_	*P*6_3_
*a*, *b*, *c* (Å)	72.03, 72.03, 98.04	72.05, 72.05, 98.03	72.05, 72.05, 98.03	72.05, 72.05, 98.03	72.05, 72.05, 98.03	72.05, 72.05, 98.03	72.05, 72.05, 98.03	72.05, 72.05, 98.03	72.05, 72.05, 98.03	72.05, 72.05, 98.03
α, β, γ (°)	90.0, 90.0, 120.0	90.0, 90.0, 120.0	90.0, 90.0, 120.0	90.0, 90.0, 120.0	90.0, 90.0, 120.0	90.0, 90.0, 120.0	90.0, 90.0, 120.0	90.0, 90.0, 120.0	90.0, 90.0, 120.0	90.0, 90.0, 120.0
Mosaicity (°)	0.034	0.032	0.035	0.033	0.034	0.032	0.032	0.035	0.032	0.035
Total No. of reflections	677419	256387	256087	256628	256062	256630	256055	255276	256440	256470
Unique reflections	107347	45917	46416	46664	46735	46757	46744	46507	46252	45650
Multiplicity	6.3	5.6	5.5	5.5	5.5	5.5	5.5	5.5	5.5	5.6
〈*I*/σ(*I*)〉	7.3 (1.1)	12.1 (3.6)	12.1 (3.6)	12.5 (3.5)	12.0 (3.4)	11.0 (3.2)	12.1 (3.4)	12.2 (3.3)	11.9 (3.3)	12.4 (3.4)
Completeness (%)	99.8 (99.5)	97.5 (94.9)	98.6 (96.6)	99.1 (97.2)	99.3 (97.6)	99.3 (97.6)	99.3 (97.7)	98.8 (95.6)	99.6 (92.0)	99.7 (92.6)
*R*_meas_ (%)	14.3 (97.4)	10.0 (33.7)	9.9 (33.3)	9.5 (33.4)	10.0 (34.7)	10.9 (37.0)	9.8 (34.7)	9.7 (35.0)	10.0 (36.2)	9.7 (35.6)
*R*_p.i.m._ (%)	4.1 (30.3)	3.0 (12.4)	3.0 (12.1)	2.9 (13.1)	3.4 (14.4)	3.4 (14.4)	3.2 (14.4)	3.2 (13.1)	3.3 (14.0)	3.2 (13.0)
CC_1/2_	99.7 (64.6)	99.6 (92.6)	99.6 (92.6)	99.7 (93.0)	99.6 (92.0)	99.5 (90.4)	99.6 (91.9)	99.6 (91.9)	98.2 (96.0)	97.0 (93.9)
Overall *B* factor from Wilson plot (Å^2^)	21.1	23.9	23.9	24.0	24.0	23.9	24.0	23.9	23.9	23.9
Isa	16.3	12.5	13.1	13.8	13.0	11.4	13.1	13.5	13.5	13.7

**Table:** (*b*) 10% transmission (0.57 MGy total absorbed dose).

	13500 eV	7114 eV	7116 eV	7118 eV	7120 eV	7122 eV	7124 eV	7126 eV	7128 eV	7130 eV
Diffraction source	BL14.1, BESSY II									
Detector	PILATUS3 S 6M									
Temperature (K)	100									
Wavelength (Å)	0.9184	1.74282	1.74233	1.74184	1.74135	1.74086	1.74037	1.73988	1.73939	1.73891
Crystal-to-detector distance (mm)	211	141	141	141	141	141	141	141	141	141
Rotation range per image (°)	0.1	0.1	0.1	0.1	0.1	0.1	0.1	0.1	0.1	0.1
Total rotation range (°)	200	200	200	200	200	200	200	200	200	200
Exposure time per image (s)	0.1	0.1	0.1	0.1	0.1	0.1	0.1	0.1	0.1	0.1
Resolution (Å)	38.53–1.34 (1.42–1.34)	48.98–1.87 (1.92–1.87)	48.98–1.87 (1.92–1.87)	48.98–1.87 (1.92–1.87)	48.98–1.87 (1.92–1.87)	48.98–1.87 (1.92–1.87)	48.98–1.87 (1.92–1.87)	48.98–1.87 (1.92–1.87)	48.98–1.87 (1.92–1.87)	48.98–1.87 (1.92–1.87)
Space group	*P*6_3_	*P*6_3_	*P*6_3_	*P*6_3_	*P*6_3_	*P*6_3_	*P*6_3_	*P*6_3_	*P*6_3_	*P*6_3_
*a*, *b*, *c* (Å)	72.02, 72.02, 98.00	72.02, 72.02, 98.00	72.02, 72.02, 98.00	72.02, 72.02, 98.00	72.02, 72.02, 98.00	72.02, 72.02, 98.00	72.02, 72.02, 98.00	72.02, 72.02, 98.00	72.02, 72.02, 98.00	72.02, 72.02, 98.00
α, β, γ (°)	90.0, 90.0, 120.0	90.0, 90.0, 120.0	90.0, 90.0, 120.0	90.0, 90.0, 120.0	90.0, 90.0, 120.0	90.0, 90.0, 120.0	90.0, 90.0, 120.0	90.0, 90.0, 120.0	90.0, 90.0, 120.0	90.0, 90.0, 120.0
Mosaicity (°)	0.038	0.034	0.036	0.039	0.036	0.036	0.035	0.034	0.040	0.037
Total No. of reflections	729013	254184	253719	253945	253868	253249	252853	252775	252820	252852
Unique reflections	126860	46429	45553	46060	45543	46092	46486	46507	45577	46157
Multiplicity	5.7	5.5	5.6	5.5	5.6	5.6	5.4	5.4	5.5	5.5
〈*I*/σ(*I*)〉	8.0 (1.1)	13.4 (4.6)	14.7 (5.1)	15.0 (5.0)	14.2 (4.9)	14.3 (5.2)	13.8 (5.1)	13.8 (5.2)	14.9 (5.5)	14.0 (5.3)
Completeness (%)	99.2 (98.2)	98.8 (97.0)	96.9 (94.1)	98.0 (95.6)	96.9 (94.0)	98.1 (95.6)	98.9 (97.3)	97.9 (95.6)	97.0 (93.9)	98.2 (96.1)
*R*_meas_ (%)	12.4 (108.3)	9.2 (26.9)	8.5 (25.7)	8.2 (25.0)	8.8 (26.0)	8.6 (23.0)	9.0 (22.7)	9.0 (22.9)	8.4 (21.9)	8.9 (22.5)
*R*_p.i.m._ (%)	3.7 (33.7)	2.9 (10.0)	2.7 (10.0)	2.6 (10.2)	2.8 (9.6)	2.8 (8.2)	3.0 (7.6)	3.0 (7.7)	2.9 (7.6)	3.0 (7.7)
CC_1/2_	99.7 (55.3)	99.6 (95.2)	99.6 (95.8)	99.7 (96.2)	99.6 (95.9)	99.6 (96.0)	99.6 (95.8)	99.6 (95.9)	99.7 (96.4)	99.6 (95.9)
Overall *B* factor from Wilson plot (Å^2^)	20.0	23.3	23.4	23.2	23.3	23.1	23.1	23.0	23.0	23.0
Isa	18.4	12.2	13.0	13.7	12.8	13.0	12.2	12.1	13.5	12.4

**Table:** (*c*) 100% transmission (2.70 MGy total absorbed dose).

	13500 eV	7114 eV	7116 eV	7118 eV	7120 eV	7122 eV	7124 eV	7126 eV	7128 eV	7130 eV
Diffraction source	BL14.1, BESSY II									
Detector	PILATUS3 S 6M									
Temperature (K)	100									
Wavelength (Å)	0.9184	1.74282	1.74233	1.74184	1.74135	1.74086	1.74037	1.73988	1.73939	1.73891
Crystal-to-detector distance (mm)	211	141	141	141	141	141	141	141	141	141
Rotation range per image (°)	0.1	0.1	0.1	0.1	0.1	0.1	0.1	0.1	0.1	0.1
Total rotation range (°)	220	200	200	200	200	200	200	200	200	200
Exposure time per image (s)	0.1	0.1	0.1	0.1	0.1	0.1	0.1	0.1	0.1	0.1
Resolution (Å)	38.56–1.36 (1.40–1.36)	38.50–1.87 (1.92–1.87)	38.50–1.87 (1.92–1.87)	38.50–1.87 (1.92–1.87)	38.50–1.87 (1.92–1.87)	38.50–1.87 (1.92–1.87)	38.50–1.87 (1.92–1.87)	38.50–1.87 (1.92–1.87)	38.50–1.87 (1.92–1.87)	38.50–1.87 (1.92–1.87)
Space group	*P*6_3_	*P*6_3_	*P*6_3_	*P*6_3_	*P*6_3_	*P*6_3_	*P*6_3_	*P*6_3_	*P*6_3_	*P*6_3_
*a*, *b*, *c* (Å)	72.15, 72.15, 97.98	72.02, 72.02, 97.84	72.02, 72.02, 97.84	72.02, 72.02, 97.84	72.02, 72.02, 97.84	72.02, 72.02, 97.84	72.02, 72.02, 97.84	72.02, 72.02, 97.84	72.02, 72.02, 97.84	72.02, 72.02, 97.84
α, β, γ (°)	90.0, 90.0, 120.0	90.0, 90.0, 120.0	90.0, 90.0, 120.0	90.0, 90.0, 120.0	90.0, 90.0, 120.0	90.0, 90.0, 120.0	90.0, 90.0, 120.0	90.0, 90.0, 120.0	90.0, 90.0, 120.0	90.0, 90.0, 120.0
Mosaicity (°)	0.064	0.050	0.053	0.042	0.045	0.044	0.046	0.055	0.048	0.057
Total No. of reflections	772936	253053	253260	253187	252837	252547	252729	252193	252691	252370
Unique reflections	122036	45792	45927	46444	46659	46732	46731	46588	46563	46060
Multiplicity	6.3	5.5	5.5	5.4	5.4	5.4	5.4	5.4	5.4	5.5
〈*I*/σ(*I*)〉	13.8 (1.1)	21.6 (11.6)	23.2 (11.7)	23.1 (10.7)	23.9 (11.2)	23.3 (10.8)	26.0 (9.4)	24.5 (8.4)	25.1 (7.6)	24.2 (6.9)
Completeness (%)	99.5 (98.8)	97.5 (94.9)	97.8 (95.4)	98.9 (97.2)	99.4 (98.0)	99.5 (98.1)	99.5 (98.2)	99.2 (95.9)	99.2 (97.5)	98.1 (93.7)
*R*_meas_ (%)	6.5 (167.7)	6.8 (11.4)	6.1 (11.0)	5.9 (12.1)	5.7 (11.3)	5.9 (11.7)	4.9 (13.8)	5.2 (15.5)	4.9 (17.1)	5.1 (19.6)
*R*_p.i.m._ (%)	1.8 (47.9)	2.0 (3.7)	1.8 (3.7)	1.8 (4.0)	1.8 (3.9)	2.00 (4.2)	1.9 (5.0)	2.0 (5.7)	1.9 (6.0)	1.9 (6.7)
CC_1/2_	99.9 (39.4)	99.7 (98.8)	99.7 (98.9)	99.8 (98.8)	99.8 (98.9)	99.8 (98.8)	99.9 (98.4)	99.9 (98.1)	99.9 (97.6)	99.9 (97.0)
Overall *B* factor from Wilson plot (Å^2^)	25.2	25.9	25.9	25.9	25.9	26.0	26.0	26.1	26.1	26.1
Isa	21.7	12.65	14.1	14.8	15.1	14.7	18.5	17.9	19.6	19.6

**Table 2:** Structure solution and refinement Values in parentheses are for the outer shell.

	13.5 keV, 5% transmission	13.5 keV, 10% transmission	13.5 keV, 100% transmission
Resolution range (Å)	38.54–1.42 (1.47–1.42)	38.53–1.34 (1.39–1.34)	38.55–1.36 (1.41–1.36)
Completeness (%)	99.80 (99.76)	99.23 (99.61)	99.46 (99.97)
No. of reflections			
Working set	54328	64525	61969
Test set	5418	6395	6177
Final *R*_cryst_ (%)	18.50	18.52	18.08
Final *R*_free_ (%)	20.82	21.48	19.92
No. of non-H atoms			
Protein	2322	2341	2370
Ligand	16	8	10
Water	318	340	351
Total	2656	2689	2731
R.m.s. deviations			
Bond lengths (Å)	0.005	0.003	0.007
Angles (°)	0.84	0.61	0.96
Average *B* factors (Å^2^)			
Overall	21.36	19.34	22.74
Protein	19.70	17.81	21.30
Ligand	19.10	20.34	24.17
Water	33.56	29.82	32.39
Ramachandran plot			
Most favoured (%)	97.88	97.88	98.21
Allowed (%)	2.12	2.12	1.79
Disallowed (%)	0.00	0.00	0.00

**Table 3:** Interatomic distances (in Å) of the metal-binding sites in diFe-SulE

	Fe-1	Fe-3	Fe-2	Fe-4
Fe–OE1 Glu20	2.18	2.19		
Fe–OE2 Glu20	2.15	2.12		
Fe–OE1 Glu53	2.06	2.07		
Fe–ND1 His56	2.19	2.24		
Fe–OE1 Glu126	2.02	2.04		
Fe–O (OH)	2.11	2.10		
Fe–OE2 Glu53			2.18	2.28
Fe–OE2 Glu92			1.90	1.94
Fe–OE1 Glu95			2.25	2.26
Fe–OE2 Glu126			2.13	2.16
Fe–O (OH)			1.87	1.85
Fe–O (H_2_O)			2.17	2.15
